# Supplementary material for: Are carbon emissions trading and green financial instruments synergistic? -Comprehensive quantitative research based on content analysis
Source: PLoS One. 2024 Mar 7;19(3):e0298601. doi: 10.1371/journal.pone.0298601 (PMC10919601; doi:10.1371/journal.pone.0298601)
Supplement: S1 Data — (ZIP) [file pone.0298601.s001.zip › Data/地级市Z_F工作报告.zip!/╔╜╬≈/╜·╓╨╩╨/└┤╫╘╣1⁄2╓┌║┼í╛┬φ┐╦╩2╛▌═°í┐_2017.docx]

各位代表：

现在，我代表市人民政府向大会作报告，请予审议，并请政协委员和其他列席人员提出意见。

一、2016年工作回顾

刚刚过去的2016年，是全面实施“十三五”规划的起步之年，是全市经济社会发展成效显著、成果丰硕的一年。一年来，我们认真贯彻落实中央、省一系列决策部署，按照省委“一个指引、两手硬”重大思路和要求，在市委的坚强领导和市人大、市政协的监督支持下，围绕“两个全面”奋斗目标，坚持创新驱动、转型升级主基调，主动作为，攻坚克难，经济发展稳中有进、稳中向好，社会事业全面进步，实现了“十三五”良好开局。

（一）经济发展在强服务、增后劲中取得新成绩。强化经济运行调度，逐月研判监测，实行精细管理、联审代办等措施，持续推进项目提质增效加速，固定资产投资增幅连续保持全省领先；扎实开展帮扶活动，延时扩面组织“干部入企服务”，深入推进受理项目和待落地项目“两个大起底”，以有力有效的举措实现了提质进位新进步。2016年，主要经济指标总体保持全省中上游水平，地区生产总值增长5.1%，规模以上工业增加值增长5%，固定资产投资增长4.8%，社会消费品零售总额增长7.5%，一般公共预算收入增长0.6%，外贸进出口额增长8.2%，城、乡居民人均可支配收入分别增长5.9%和6%，实现了省委省政府“下半年好于上半年，为明年进一步好转奠定坚实基础”的目标要求。其中，地区生产总值、规模以上工业增加值、外贸出口额3项总量实现进位，地区生产总值、规模以上工业增加值、固定资产投资、一般公共预算收入4项增幅居全省前列。

（二）产业转型在调结构、促升级中迈出新步伐。坚持煤电一体化、装备高端化、农业现代化、旅游集团化、业态新型化，推动产业结构优化升级。吉利新能源汽车、东方希望铝、博创纳米材料、中鼎物流园、乌金山李宁国际滑雪场等转型标杆项目投产运行，4个低热值煤发电、晋能光伏电池、杉杉奥特莱斯等项目加快建设；启动创建国家全域旅游示范区，推进产品、管理、营销、业态“四个升级”，全年接待游客6357万人次、位居全省第一，旅游总收入658亿元、位居全省第二，分别增长26%、28%；粮食生产再获丰收，总产量18.4亿公斤，国家级现代农业示范区加快建设。全年非煤产业增加值占规上工业43.2%、提高5.4个百分点；高新技术产业占规上工业3.9%，提高0.6个百分点。

（三）城乡面貌在夯基础、抓管理中发生新变化。市城区连续六年实施“百亿城建重点工程”，市政道路当年投资、建成里程创历史之最。东南外环、迎宾街和锦纶路北段改造等快速互通项目主线通车，晋中太原城际铁路2号线加快建设；中小学示范性综合实践基地、烈士陵园、晋中大剧院建成投入使用，市博物馆、科技馆、图书馆、工人文化宫、第一人民医院等公共服务项目进展顺利，社火公园建成开园；王湖、侯方基本完成整村拆除。创新城市管理，加强市容环境综合整治，在全省率先实行城乡一体第三方考评，昔阳县成功创建国家卫生县城，寿阳县成功创建省级园林县城，大寨镇成功创建国家卫生乡镇并入选国家特色小镇。我市被国家国防动员委员会命名为“全国人民防空先进城市”。

（四）改革创新在抢机遇、破难题中实现新突破。积极推进供给侧结构性改革，实行煤炭减量生产和扩大先进产能相结合，原煤产量下降15.6%，煤炭产业增加值增长2.1%；商品房待售面积比年初减少8.6%，去化周期保持在10个月以内；金融机构表外融资降低11.1%；减轻企业负担46.8亿元；交通、水利等基础设施完成投资291.8亿元，增长24%。紧紧抓住国务院表彰、“国字号”改革试点等机遇积极先行先试，各项深化改革年度任务基本完成，农村土地确权、政府购买服务等改革走在全省前列；山西转型综改示范区晋中区、山西“农谷”建设筹备工作顺利推进，108廊带示范区整体建设方案出台，重大标志性工程开工建设；2个市本级、1个县级项目入选财政部PPP项目示范库。晋中银行太原分行和北京、上海两个财富中心投入运营，资产规模实现翻番。

（五）民生事业在补短板、上水平中得到新改善。

全力打好脱贫攻坚开局之战，精准实施六大攻坚工程，151个贫困村退出、4.9万贫困人口脱贫。全面实施大学生就业创业促进计划，晋中大学生创业园投入运营，建成7个创业孵化基地，全市城镇新增就业3.5万人，农村劳动力转移就业3.47万人，城镇登记失业率控制在4.2%以内。扎实推进机关事业单位养老保险制度改革和城乡居民基本医疗保险整合，居民大病保险实现参保全覆盖，并在全省率先实现医院直接结算。城乡义务教育均衡发展工作走在全国前列，各级各类教育协调发展政策制度在全省推广。基层卫生计生融合整合和乡村医疗卫生机构“五统一”管理走在全省前列。“国家公共文化服务体系示范区”建设、全省首家工业遗产保护利用项目“晋华1919”进展顺利，全民健身活动广泛开展。全年各类安全生产事故起数、死亡人数同比下降0.36%和2.87%，未发生重大以上安全生产事故。

（六）政府建设在转作风、提效能中焕发新气象。自觉接受人大法律监督和政协民主监督，办理人大代表意见、建议和议案248件，政协委员提案282件，满意率均达到99%以上。严格依照法定权限和程序履行职责，完善政府法律顾问制度，进一步加强对规范性文件的合法性审查。严格落实“13710”工作制度，建立电子台账，细化工作流程，提高落实质量。建成 “市民之家”，政务服务平台和公共资源交易平台投入运行。强化督查考核，紧盯重点事项，一周一清单推进重点工作落实。切实提高行政效能，马上就办、真抓实干的工作作风体现在加快重点工程建设的速度上，体现在推动重点工作开展的进度上，体现在解决难点问题的力度上。严格落实党风廉政建设主体责任和“一岗双责”，各级干部的责任意识、服务意识和廉洁意识进一步增强，干事创业的氛围日益浓厚。

各位代表，过去一年取得的成绩来之不易。这是省委、省政府关心关怀、坚强领导的结果，是市委统揽全局、正确领导的结果，是市人大、市政协大力支持、有效监督的结果，是全市广大干部群众团结拼搏、苦干实干的结果。在此，我代表市人民政府，向全市人民，向各民主党派、工商联和无党派人士，向各位人大代表、政协委员，向驻地人民解放军、武警官兵、公安民警和国家、省驻地单位，向所有关心支持晋中改革发展的朋友们，表示崇高的敬意和衷心的感谢！

在充分肯定成绩的同时，我们也要清醒地看到存在的问题，主要是：经济总量不大，产业结构不优，总体发展水平与区位优势不相匹配；经济增长缺乏大项目支撑，发展后劲有待持续加强；城乡发展不平衡，脱贫攻坚任务艰巨，社会事业亟需补齐短板；资源环境约束加剧，环境污染问题突出；安全生产隐患不容忽视，社会治理仍需加强和创新；政府职能需要进一步转变，一些干部不作为、不敢为、不会为，一些部门和地方服务意识不强、行政效率不高、工作落实不力，等等。对这些问题，我们必须高度重视，采取更加有力的措施，认真加以解决。

二、2017年工作安排

2017年是实施“十三五”规划的重要一年，是供给侧结构性改革和转型综改的深化之年，是我市全面挺进全省第一方阵的首战之年。政府工作的总体要求是：深入学习贯彻习近平总书记系列重要讲话精神和省委治晋理政新方略，统筹推进“五位一体”总体布局，协调推进“四个全面”战略布局，认真贯彻落实省第十一次党代会和省委十一届二次全会暨经济工作会议精神，按照市四次党代会和市委四届二次全会暨经济工作会议总体部署，坚持新发展理念，坚持稳中求进工作总基调，坚持深化供给侧结构性改革与深化转型综改试验区建设有机结合，坚持以提高质量和效益为中心，全面实施创新驱动、转型带动、改革推动、项目拉动四大战略，突出抓好转型升级、改革创新、项目建设、城乡发展、脱贫攻坚、环境改善、民生保障、政府建设八个重点，全力促进经济稳步向好、民生不断改善和社会和谐稳定，为全面挺进全省第一方阵开好局、起好步，以优异成绩迎接党的十九大胜利召开。

主要预期指标是：地区生产总值增长6%，规模以上工业增加值增长3.2%，全社会固定资产投资增长目标根据新的统计口径设置，社会消费品零售总额增长7%，一般公共预算收入增长4%，城乡居民人均可支配收入分别增长7%和7%以上，居民消费价格涨幅控制在3%左右，城镇登记失业率控制在4.2%以内。

约束性指标是：资源节约方面，万元地区生产总值能耗下降3.2%，万元地区生产总值二氧化碳排放量下降3.9%，万元地区生产总值用水量下降3.2%。环境质量方面，市城区PM2.5浓度、空气质量优良天数比例达到省考核要求；11个国、省考断面中，Ⅲ类以上断面比例达到72.73%以上，劣Ⅴ类断面比例控制在27.27%以下；二氧化硫、化学需氧量、氨氮、氮氧化物减排不折不扣完成省下达任务。民生改善方面，农村贫困人口脱贫5.9万人，城镇棚户区住房改造7100套。

围绕上述目标，按照全面挺进全省第一方阵指标体系要求，力争完成的更好，重点抓好以下八方面工作：

（一）持续深化供给侧结构性改革，努力在构建晋中特色现代产业体系上取得突破。把深化供给侧结构性改革作为加快产业转型升级的重大机遇，以改善供给结构为主攻方向，用好资源优势，扩大基础优势，放大区位优势，加快培育形成新的增长动力。

坚决完成“三去一降一补”任务。在去产能上，坚持把去产能与提高先进产能占比、优化产业结构结合起来，建立优胜劣汰长效机制和煤炭企业转型促进机制，支持灵石煤焦电铝硫化铁循环项目发展，全面完成省定煤炭去产能年度任务；积极推进工业园区增量市场售电侧改革，建立大用户直购电和10千伏高新技术项目直购电申报绿色通道。在去库存上，推动工业品去库存，实行甲控（供）管理，协调推动我市工业企业、重点项目产需对接；继续落实促进农民工进城、扩大货币化安置、刺激改善型消费等房地产去库存政策，遏制投资投机性购房。在去杠杆上，把降低企业负债率作为重中之重，引导企业通过银行间市场发债改变融资结构，发展股权融资，对因行业周期性波动面临暂时困难的企业、战略性新兴产业领域的成长型企业和居于过剩行业前列的关键性企业，优先支持去杠杆。在降成本上，坚持降成本与强管理相结合，落实好制度性交易、中介服务、税费、融资、用电、物流等方面降低实体经济企业成本的政策措施，强化企业精细化生产和对标管理，增强盈利能力。在补短板上，重点在脱贫攻坚、基础设施、公共服务、生态环保等方面，加大投入、打牢基础。

加快做大做强文化旅游产业。以创建国家全域旅游示范区为抓手，围绕打造千亿旅游产业目标，着力把文化旅游业培育成战略性支柱产业。高标准完成全域旅游规划编制工作。坚持政府主导，发挥专家作用，突出指导性、针对性和可操作性，抓紧制定出台全域旅游发展建设规划。加快体制机制改革创新。3月底前市县两级完成旅发委组建和挂牌，6月底前完成列入全省重点改革名录的10处景区（景点）改革任务，年底前完成全市旅游管理体制和景区经营机制改革。完善旅游产业体系。坚持“旅游业+”发展思路，把握“安、顺、诚、特、需、愉”六字要诀，围绕构建晋商家国文化和太行山生态度假两大旅游产品体系，大力发展生态、度假、康养、乡村、红色、体育旅游等新业态，建设一批特色文化旅游园区、生态庄园、省级休闲旅游度假区和红色旅游景区，打造一批特色旅游线路；推动旅游与一二三产业融合发展，实现旅游产业全域辐射带动；开展旅游扶贫工作，充分发挥旅游富民功能；积极申报全省旅游发展大会主办权，办好平遥国际摄影展和电影展、中蒙俄万里茶路市长峰会等节庆活动；加强与华侨城等旅游大集团合作，实施好昔阳大寨、榆社云竹湖、和顺鹊桥生态文化园、左权百里红色走廊、介休张壁古堡等项目建设；加快王家大院5A景区创建步伐。强化服务保障。进一步完善全市旅游公共服务设施，规范旅游市场秩序，提升服务质量和管理水平，确保全年接待境内外游客7350万人次、旅游总收入760亿元。

加快发展高端装备制造业。以吉利山西新能源汽车项目为龙头，全面推进新能源汽车装备制造园区建设，年内生产轿车10万台、新能源商用车500台，年产值达到80亿元；积极引进汽车零部件配套企业，构建新能源汽车产业链。纺机产业突出龙头企业做强成套主机、配件企业做精配套产品两大重点，完善产业配套合作机制，提升市场拓展能力，全年产值突破 24亿元。液压产业重点推进太重榆液产品试验检测中心投产运行，加快高行液压高铁导轨板生产线项目建设，推动方盛液压由液压元器件向生产液压成套装备升级，全年产值突破14亿元。

加快培育清洁能源产业。加大煤层气开发利用力度，抓紧出台加快产业发展的工作方案；完成煤层气资源及矿权调查摸底，加快已设立探矿权区块资源勘探；落实好国家和省各项支持政策，加强对接协调，加快中联、中石化煤层气和漾泉蓝焰左权未利用井的开发利用，推进凯嘉煤层气发电等项目，全年煤层气抽采量力争达到5.5亿立方米，利用量3亿立方米以上。鼓励发展可再生能源，推进一批风力、光伏等发电项目，抓好寿阳1GW光伏、晋能高效光伏电池及组件项目建设与投产达效。

加快提升现代农业。全面贯彻中央1号文件精神，以推进农业供给侧结构性改革为主线，加快培育现代农业发展新动能。调整优化产业结构，在稳定粮食生产的同时，发展设施蔬菜1万亩，优质水果1万亩，优质高产干果3万亩，中药材10万亩。大力培育新型经营主体，规范提升省市县示范合作社100个，示范家庭农场10个。促进一二三产融合发展，扶持壮大8个农产品加工产业集聚区和10大产业集群，打造10个领军企业、100个骨干企业、200个小微企业集群，农产品加工业销售收入突破200亿元，祁县酥梨、昔阳双孢菇等特色农产品出口创汇突破1500万美元；以2个国家级、15个省级、55个市级休闲农业与乡村旅游示范点为重点，全面推进提质升级，休闲农业营业收入达到10亿元。推行绿色生产方式，完成无公害农产品、绿色食品、有机农产品认证30个以上，认定面积22万亩。推进农作物秸秆综合利用，综合利用率力争达到90%以上。加快国家现代农业示范区建设，围绕粮、菜、果、牧及休闲农业五大产业，重点在6个创建县区打造国家现代农业示范区核心片区。

加快壮大特色产业。玻璃器皿产业依托祁县“中国玻璃器皿之都”品牌，发挥祁玻集团集聚效应，加快行业整合壮大，出台玻璃器皿产品地方标准，完成国家级玻检中心实验室等级验收，积极争取10月份筹办首届中国玻璃器皿节暨中国日用玻璃协会2017年年会。玛钢铸造产业以太谷胡村玛钢铸造园为突破，全面启动“气化玛钢”，重点推进华北铸造物流城等项目，完善研发会展中心功能，办好山西玛钢铸造产品展示交易会，建成国家级玛钢铸造检验检测中心。医药产业以高成长性医药企业为突破口，支持广誉远、德元堂等企业进一步做强做大；加快广生普鲁兰多糖、振东安特中药家护用品项目前期工作，力争年内开工建设。特色食品围绕食品企业规模化、集约化发展，重点抓好伊利、统一、娃哈哈项目一期达产达效和二期前期工作，加快晋汉生物科技一期项目建设，年内建成投产。

加快改造提升传统产业。认真落实省政府关于推进企业技术改造部署要求，制定出台具体政策措施，聚焦新技术、新产品、新工艺、新装备、新模式、新业态，推动企业技改扩规、提质增效。加大煤、焦、冶、化、电等行业技术改造力度，延伸产业链、提升价值链，重点推进灵石和介休两个千万吨级焦化集聚区、东方希望铝系工业园二期、阳煤寿阳乙二醇、阳煤昔阳化工园区聚氯乙烯、烧碱二期以及4个低热值煤发电等项目的技改和扩产。

加快实施品牌战略。发挥标准对品牌创新的引导作用，组建玻璃器皿等重点产业标准专业委员会，引深标准化试点示范项目建设，鼓励企业或社团组织发布执行联盟标准、团体标准。加强质量管理，整合提升全市质量检验检测能力和服务平台，筹建晋中市质量与品牌发展研究机构，鼓励“榆次液压”“太谷玛钢”“平遥牛肉”创建全国知名品牌示范区，继续实施“市长质量奖”，开展精益管理和标准创新分级评定，在20家重点企业实行首席质量官制度。推动品牌孵化增值，通过品牌培育和传播，打造具有晋中城市名片效应的产业集群，塑造晋中质量和晋中品牌良好形象。

（二）持续抓好事关全局的关键性改革，努力在转型综改上取得突破。把改革创新作为今年政府工作的重要任务，认真落实深化改革各项任务，积极探索、先行先试，着力破解制约经济发展的体制机制问题。

全面推进山西转型综改示范区晋中区建设。坚持转型为纲、产业为王、创新为上、改革为要，突出重点，统筹推进，打造全市转型综改“主战场”。按照“政企分责型”模式，依托晋中经济技术开发区管委会设立山西转型综改示范区晋中区管委会，与省同步挂牌运行。加快出台相关政策制度。抓紧推进土地利用总体规划调整、规划编制、招商引资等工作，4月份全面启动起步区建设，先行实施山西半导体产业园、晋中通用航空产业园等20个重点产业、基础设施项目，尽快形成示范带动效应。推进示范区和县级开发区联动发展，在示范区晋中区设立对全市开发区（园区）统一协调服务的综合平台，构建“1+10”联合体，实现规划统筹、产业协同、资源共享、服务联动、合作共赢。推进全市现有开发区（园区）整合扩区，按照每县（市）设立一个开发区的要求，祁县、介休两个省级开发区要积极推进整合、改制、扩区、调规，加快发展壮大；太谷、平遥、灵石、昔阳四县要采取整合、共建、一区多园等模式，统一进行规划建设。各县（市）开发区要在5月底前完成总体布局规划编制，同步推进“三化三制”和统计制度改革、社会职能剥离、目标责任考核等各项工作。将108廊带示范区建设融入转型综改示范区建设和开发区改革创新工作中，启动108国道市城区段人居环境综合改善工程，完成项目前期工作，力争年内开工建设；推进108科技产业园等重点项目建设，年内取得实质性进展。

加快打造山西“农谷”。 以太谷县为核心，以全县域、全要素、全产业方式，聚焦“研发高地和产业洼地”两大核心功能，全力推进山西“农谷”建设。加快制定管委会三定方案和事权划分方案，3月份挂牌运行。加快规划编制，3月底前完成山西农谷建设总体规划和基础设施及农科城、北方林果科技园等专项规划。借鉴省内外各类园区和省转型综改示范区的经验做法，制定出台农谷发展优惠政策，吸引人才、产业、科技、资金等要素集聚。以创建全国功能农业综合示范区为目标，重点建设山西功能农业研究院、功能食品研究院、国家功能食品检验检测中心，推进药食同源产品开发，为中高端市场提供具有保健功能的食品。今年重点实施总投资196亿元、年度投资41亿元的27个项目，完成园区路、水、电、气等基础设施建设，基本建成“农谷”雏形，引领示范和辐射带动全市、全省特色现代农业发展。

深化国资国企改革。认真贯彻落实全省国有资产监督管理工作会议精神，加强党对国有企业的领导，推进党的领导融入企业法人治理结构、党的建设与生产经营深度融合，切实发挥国有企业党组织领导核心和政治核心作用。创新国有资产监管，明确国有资产出资人职责定位，实现 “管企业”为主向 “管资本”为主转变；继续推动财务等重大信息公开常态化、制度化，引深经营绩效考评，进一步完善资产、财务等管理制度，提升国有资本使用效益。稳步推动国企改革，晋华、锦纶、轻机三户政策性破产企业在完成职工安置、财产处置、学校医院幼儿园剥离移交的基础上，力争终结破产法律程序；完成药材公司职工安置及财产处置，启动市属国有企业9户厂办大集体改革；完成山西佳新国旅有限公司国有股权转让，推动市押运护卫中心开展混合所有制改革，加快推进商粮系统18户企业改革。

深化财税和金融体制改革。推动财政预算改革和全过程绩效管理，加大预算执行监管和支出优化整合力度，加快推进市县两级财政事权和支出责任划分，探索在部分领域引入竞争性使用财力机制。加大金融工作力度，继续对全市金融机构考核激励，强化政银企合作，确保全年新增贷款高于去年；做强做大地方金融机构，晋中银行资产规模突破800亿元，左权、榆社2家支行年内挂牌；加快推进农信社改制农商行步伐，年内完成太谷、开发区、榆社3家农信社改制；落实好企业上市奖励政策，年内新增新三板上市公司 3户。进一步做好非法集资风险防控工作。

深化科技和人才发展体制机制创新。完善科技计划管理等配套政策，鼓励高校技术人才和大学生创办企业，重点扶持和培育高成长性的科技型中小微企业60家，全年新增10家高新技术企业；提升全市产业整体创新水平,重点扶持成果转化项目20个，实现全市技术合同交易额12亿元、有效发明专利拥有量460件；创新科技服务方式，鼓励企业与高校院所共同承担科技重大专项和重点研发项目，积极落实加计扣除、税收减免、专利资助等政策。坚持人才培养和引进并举，落实以增加知识价值为导向的分配政策，从成果转化、表彰奖励、政府投入等方面形成鲜明导向，吸引高层次人才；全面落实用人单位自主权，取消机关事业单位控编进人卡和进人计划卡，打通政企、事企人才双向流动通道，构建全新的人才自主流动机制。

深化农村综合改革。细化落实农村土地所有权、承包权、经营权三权分置办法，全面完成农村土地承包经营权确权工作，基本完成登记颁证任务；加快农村产权流转交易市场体系建设向乡村延伸，实现乡镇交易所全覆盖，在太谷、左权、榆社、寿阳4个县开展农村产权流转交易规范化建设；摸清家底，建立台账，年内全市三分之一的行政村完成清产核资；开展农村集体资产股份权能改革试点，在集体经济组织成员身份确认、股权量化和资产管理等方面积极探索、取得经验；推进国有林场和集体林权制度改革，探索建立碳汇造林机制和新型营造林投融资机制；以平遥、左权为试点，深化供销社综合改革，加快构建经营性服务和公益性服务相融合的合作经济组织体系。

各位代表，改革是发展的必由之路，创新是引领发展的第一动力。我们要坚持深化改革不动摇，坚持创新驱动不松劲，全力做好综改牵引、创新转型这篇大文章，让发展的活力不断迸发、发展的动力更加强劲、发展的空间更为广阔！

（三）持续推进重点项目建设，努力在增加有效投资上取得突破。着眼于加快转型发展，以优化投资结构、提高投资效益为重点，狠抓项目建设。目前，市政府已制定了《扶持转型项目和优势企业实施方案》《培育壮大战略性新兴产业和特色产业实施意见》，并着手制定一系列配套措施，形成转型发展“2+5”政策体系，今后三年重点扶持总投资上千亿元的100个转型项目和总产值过千亿元的35户优势企业。今年实施61个转型项目，确保完成投资260亿元以上。

创新项目推进机制。建立市县两级政府领导领办转型项目机制，根据工作分工全程协调、全程服务，成立市县两级企业服务办公室，完善专业化团队代办服务机制，从市直经济部门和部分自收自支事业单位优选100人左右，与市县入企帮扶小组统筹整合，组建30个专业化服务小组，选优配强组长，市经信委归口管理，经专业化培训后，为转型项目和优势企业全程提供“保姆式”代办服务，3月底前完成组建，4月份开展工作。实行重点项目动态调整机制，建立预备项目库，制定政府投资项目滚动投资计划，9月底视项目推进情况进行动态调整。建立重要问题解决定期通报制度，每月梳理上报需省市级层面解决的问题，统筹协调解决，市政府月度县（区、市）长例会通报进展情况。

创新招商引资办法。围绕精准招商、定向招商和提高项目成熟度，主动融入京津冀，承接好东部省份加工贸易产业转移，创新项目策划包装，制作“招商地图”，在落实我省与天津等地开展的全面深化合作中争取更多大项目好项目。创新招商方法，实施政府购买招商项目专业化服务，充分调动社会各方面力量参与招商引资的主动性和积极性。创优项目落地条件，根据县域产业规划，研究探索出台差异化招商引资优惠政策；鼓励发展合作共建、托管等模式，探索零土地指标招商，落实高用电项目用电优惠奖励等措施。完善考核体系，把招商引资工作纳入目标责任考核范围，重点考核签约项目开工率、资金到位率，并加大考核权重。全年引资总额2100亿元，签约项目落地率达到50%以上。

创新要素保障方式。资金保障上，进一步拓宽渠道，撬动更多社会资本参与项目建设。市财政预算安排2亿元专项资金，通过股权投入、项目引导、示范奖补等方式推广PPP模式；安排2亿元设立促转型增动能投资基金和促转型增动能担保资金，为转型项目和优势企业落地建设、技术改造等提供增值服务、放大担保能力。用地保障上，在完成永久性基本农田划定和土地利用总体规划调整完善的基础上，向内挖潜、盘活存量，做好城乡建设用地增减挂钩，充分利用闲置土地，缓解规划指标紧张压力；对转型项目和优势企业用地，实行土地出让金分期缴纳，试行工业用地弹性出让，最低出让期由规定的50年缩短为30年，降低前期投入成本。

（四）持续加快扩容提质，努力在新型城镇化发展上取得突破。坚持为民城建理念，以太原都市区建设为引领，传承彰显晋中特色，统筹集聚各种要素，不断完善城市功能、提升城市品位、增强辐射能力，推动解决“一市一区”问题。

强化基础建设。坚持地上地下同建、面子里子齐抓，市城区实施当年投资277亿元的百项城建重点工程。提升公共服务能力，实施经纬中学、北部新城高中、榆次区实验小学等7所公办学校新建、续建、扩建项目；市博物馆、图书馆、科技馆、殡仪馆、第一人民医院等公共服务项目投入使用；万达广场、杉杉奥特莱斯等商业服务项目建成运营。进一步完善路网体系，畅通交通出行，重点实施蕴华街拓宽、安宁街西延等13个市政道路项目，改造花园巷、定阳路等18条小街巷，加快晋中太原城际铁路2号线建设，做好1号线前期工作。强化便民服务，新建便民市场11个，停车场8个。改善居住条件，实施寇村、龙田等22个城中村改造，推进迎宾广场、晋中学院旧址等5个片区改造。优化城市环境，加快晋商公园和潇河公园三期工程建设，启动学府生态湿地公园建设，新建街旁绿地10处。科学规划利用地下空间，完成迎宾北广场、“三馆”地下空间综合利用项目，启动工人文化宫广场、榆次一中地下停车场等项目，地下空间综合利用面积达到200万平方米,增长66%；改造供水、供热危旧管网，增加燃气管道和中水管网，实施龙湖街东延、广安街等10.4公里地下综合管廊建设。

强化规划管理。坚持规划引领，编制完成城区停车场、便民市场、绿化、亮化、历史风貌保护等专项规划，启动各县市重点基础设施专项规划。引深市容环境综合整治，突出抓好街巷街景、小广告清理、车辆乱停乱放、私搭乱建、马路市场、小区环境卫生六大专项整治，完善城乡市容市貌和环境卫生考评及第三方监督管理办法，解决好城市脏乱差问题。深化城市管理综合执法体制改革，坚持权责统一、重心下移，实行权随事走、人随事调、费随事转，3月底前基本完成市城区城市执法体制改革试点工作，并在介休、左权开展试点，年底实现全市城市管理机构综合设置。全面推进“四城联创”，以争创全国文明城市为引领，统筹推进国家园林城市、国家卫生城市和全省环保模范城市创建工作。

强化城乡统筹。加快县城提档升级，完善市政基础设施和公共服务设施，全市新建改造燃气管网95公里，普及率达92.5%以上；新增集中供热面积200万平方米，普及率达94%以上；城市污水处理率提高到95.6%，垃圾无害化处理率达85%；新建绿地215万平方米，建成区绿地率达34%、绿化覆盖率达39%、人均公园绿地面积12.6平方米。推进特色小镇建设，新创建国家级省级特色小镇2个。深入实施农村人居环境改善工程，深化灵石县农村垃圾治理和介休市生活污水治理试点工作，打造市级美丽宜居示范村15个。优化城乡交通条件，开工建设晋衡高速榆次至昔阳段，加快推进左权县城至黎城下清泉段（晋冀界）新建工程、和顺董榆线一级路改造二期工程、207国道祁县境内一期工程和阳大高速铁路项目前期，做好龙城高速北延及离石至祁县高速建设协调保障工作，新建改建农村公路400公里。继续配合东山供水、中部引黄两大水网建设，实施好娘子关调水工程，启动5县（市）县域小水网建设，巩固提升8万人饮水安全标准。推进“宽带中国”示范城市创建，开展45个行政村宽带建设和改造。完成186个村农网升级改造，新建、改造配变1145台，提升区域性电力供应保障能力。

（五）持续实施精准脱贫，努力在打赢脱贫攻坚战上取得突破。以四个贫困县为主战场，按照“六个精准”要求，全力推进六大攻坚工程，全面完成二十个专项行动年度任务，确保5.9万人脱贫，284个贫困村退出，昔阳县率先摘帽。

抓好产业。坚持市场导向、因地制宜，围绕农业、光伏、旅游、电商等特色产业，在四个贫困县实施优质核桃经济林提质增效12万亩，巩固发展23万亩优质小杂粮种植，新增5万头家畜和100万只家禽养殖、50万平米菌类、0.9万亩蔬菜、7万亩中药材，区域光伏电站装机容量突破100兆瓦，扶持45个村发展乡村旅游，推广和顺县电子商务进农村模式，60%的贫困村发展电子商务应用。

用好政策。统筹运用和落实好各类扶贫政策，推动政策精准落实到村到户到人。充分发挥好产业政策的支撑作用，精准确定产业，因户对接产业，确保贫困村形成特色优势产业，贫困户在产业健康发展中稳定增收；充分发挥好社保政策的兜底保障作用，着力推动教育、医疗、文化、科技、就业、社会救助等公共服务和社会保障政策措施全面落实；充分发挥好金融政策的引导作用，全市贫困户小额贷款和强农贷款达到5亿元。

打好基础。重点从基础工作、基础设施、公共服务、社会保障等方面抓起，全面落实脱贫攻坚规划和当年行动计划，有效指导和推进工作；实施总投资3.8亿元的道路、水利、电网、人居环境等基础设施提升工程，加快改善贫困地区生产生活条件，完成易地搬迁5400人。

搞好帮扶。凝聚干部、企业、社会三方力量，落实单位包村、领导包带和工作队到村、党员干部到户、第一书记到岗的“两包三到”精准帮扶联动机制，动态建好用好精准脱贫手册，密切关注贫困家庭变化情况，实施全程管控、动态管理,及时记录贫困户相关信息,及时调整脱贫政策措施,确保精准填报、精准施策。坚持扶贫与扶志扶智相结合，促进贫困户解放思想、转变观念；充分发挥帮扶单位自身优势，配合县、乡、村三级抓好业务培训，提升贫困群众的知识水平和劳动技能，实现持续脱贫、本质脱贫。

（六）持续狠抓生态环境保护，努力在美丽晋中建设上取得突破。以提高生态环境质量为核心，实施最严格的生态环境保护制度，统筹抓好源头、过程、重点、专项和系统治理，促进全市生态环境质量不断改善。

打好铁腕治污硬仗。实施大气、水、土壤污染防治三大攻坚，强力推进铁腕治污行动，加大环保督查力度，持续改善全市环境质量。多措并举清霾。以猛药去疴的决心和壮士断腕的勇气，采取十项措施，重拳出击，全面发力。一是修订并落实市城区高污染燃料禁燃区总体规划，合理确定禁燃区；二是对市城区14个区域集中供热站进行整合，提高热电联供比例，严格执行大气污染排放特别限值；三是淘汰市城区35台361吨20蒸吨以下和县城区180台310吨10蒸吨以下燃煤锅炉；四是拆除改造环城路以内27个城中村11413台土小锅炉（1.2万户228万平方米），全部实行集中供热或清洁能源替代；五是对榆次工业园区16户23台126吨工业锅炉和怀仁村100户128台70吨土小酿造用燃煤锅炉，全部实施清洁能源或工业蒸汽替代；六是强化电力、焦化等重点行业脱硫脱硝改造；七是市城区全面禁止烟花爆竹燃放，推动禁炮立法；八是全面整治面源污染，取缔市城区露天烧烤摊点和使用散煤餐饮摊点，严禁露天焚烧垃圾；九是强力推进城区扬尘污染治理，实现所有施工工地围挡、苫盖、洒水、绿化、硬化、冲洗“六个百分百”；十是修订重污染天气应急预案，加强天气预测预警，落实应急减排措施。综合施策治水。全面推行河长制，统筹推进工业水污染防治、城镇生活污水设施提标改造和农村水环境整治，完成31项规模化养殖场农业源治理项目和散养密集区污染防治设施建设，加快潇河市城区段治理。标本兼治净土。完成土壤污染治理与修复规划，强化危险废物规范化和工业场地再利用项目管理，因地制宜推行垃圾分类制度。

打好节能降耗硬仗。重点在冶金、电力、煤炭、焦化、化工、建材等六大主要耗能行业开展“双百企业节能行动计划”，推行合同能源管理，支持企业能源管理控制中心和能源管理体系建设。实施绿色建筑行动，全市城镇规划范围内绿色建筑占新建建筑比例达到35%以上。推动交通节能，加大新能源汽车推广力度，年内建成充电桩1000个，在10个加油站完善甲醇加注设施，全市新增、更新公交车辆全部使用新能源汽车。

打好生态建设硬仗。推进省级林业生态市建设，

加快打造“双百公里林果带、千里林业生态圈”，启动新一轮退耕还林，全年新造林合格面积35万亩以上，林木绿化率增长1.5个百分点以上。启动汾河流域生态修复祁县、平遥、介休核心区项目和左权县清漳河国家级湿地公园建设，做好祁县昌源河和介休市汾河2个国家级湿地公园验收工作，推进全市80个生态庄园提质增效。推进采煤沉陷区和农村地质灾害治理，分别启动175户和327户治理搬迁项目。加大森林资源保护力度，切实做好森林防火工作。

各位代表，绿水青山就是金山银山，改善环境就是发展生产力。我们要顺应人民群众期待，回应人民群众关切，坚持不懈加强生态文明建设，久久为功狠抓生态环境保护，努力让晋中天更蓝、水更清、地更绿、环境更宜人！

（七）持续保障和改善民生，努力在提升人民群众获得感和幸福感上取得突破。坚持以人民为中心的发展思想，守住底线、突出重点、完善机制，让改革发展成果更多更公平惠及全市人民。

进一步加快发展社会事业。深入推进现代化教育强市建设，实施第三期学前教育行动计划，加强小区配套幼儿园建设，建立普惠性民办幼儿园扶持机制；统筹推进城乡义务教育一体化改革发展，深化义务教育均衡发展等6项国家级教育改革试点，启动中考制度等6项省级教育试点改革和农村中小学厕所“旱改水”工程，落实特殊教育提升计划；完成7所普通高中标准化建设评估，全市公办普通高中学校全部达到省定办学标准；落实中职学校生均拨款制度，建成10个省级职业教育实训基地，6所职业学校基础能力通过达标验收。实施健康晋中计划，巩固县级公立医院改革成果，推进8所城市公立医院综合改革；加快33所县乡级医疗卫生机构基础设施建设，做好榆次、平遥、寿阳分级诊疗试点工作；开展家庭医生签约服务，重点人群覆盖率达到60%以上；积极推进健康城市和健康村镇试点建设。深入实施全民健身国家战略，完善公共体育服务体系，做好全国青运会承办项目筹备和宣传工作，办好晋中特色品牌赛事。

进一步增强文化软实力。全面实施核心价值养成、公民素质提升、城市形象塑造、传播体系建设、公共文化惠民、文化产业升级、文艺精品创作、文化改革创新、优秀文化传承、人才队伍提质“十大工程”。以市县乡村四级公共文化设施网络为抓手，围绕实现“九化、五机制”，全面完成国家公共文化服务体系示范区创建任务。深入推进晋中国家文化生态保护实验区建设，抓好晋商文化、大寨文化和太行红色文化资源的保护利用，实施乡村文化记忆工程，建成晋中非遗展示馆。繁荣艺术创作，重点推出一批文艺精品力作。加强文物保护利用，完成“晋华1919”项目。加快文化产业发展，推动文化与旅游、科技、教育、体育、金融等产业融合，积极扶持引导文化创意、动漫游戏、数字印刷等新业态，办好“中国手艺发展大会”，启动“中国手艺小镇”建设。发展新闻出版、广播影视、版权事业，加强“扫黄打非”和文化市场整治，不断净化文化环境。

进一步提高就业和社会保障水平。积极扩大就业，组织实施好高校毕业生、返乡农民工、城镇就业困难人员等重点群体就业创业计划，推进创业孵化示范基地建设，全年新增城镇就业3.5万人。全面加强社会保障，推动机关事业单位养老保险制度改革入轨运行；实现城乡居民基本医保制度并轨，推进基本医保异地结算和支付方式改革；开展生育保险和基本医疗保险合并实施试点；提高城乡低保和特困人员救助供养标准，推动“脱贫线”和“低保线”有效衔接。开工建设保障性住房7100套，改造农村危房3627户。全面加快养老服务业发展。提高养老服务质量，关系全市48万老年人口特别是8.6万失能半失能老年人的晚年幸福，也关系他们子女工作生活，是涉及人民生活质量的大事。要抓住全国养老服务业综合改革试点这一契机，突出重点，统筹推进各项工作。3月底前编制完成“十三五”养老服务业发展专项规划，制定出台年度行动计划；抓紧编制养老服务业发展相关配套专项规划，形成完整的政策支持体系；加快居家、社区和机构养老服务设施建设，市老年养护院投入使用，新建10所城市社区养老服务中心，支持太谷县创建省级康养产业示范园区，全市新增养老床位1200张，千名老人床位数达到37张，继续保持全省领先；加快医养融合发展，积极推进老年人医养护理体系建设，在晋中一院等二级以上医疗机构设立老年病科，新建改建5个医养结合机构，完善医药报销机制，畅通老年人在养老机构看病用药报销渠道。

进一步筑牢安全生产防线。全面落实《中共中央国务院关于推进安全生产领域改革发展的意见》和《山西省安全生产条例》，严格落实“三个坚决防止”和“四铁”要求，把坚决杜绝重特大事故发生作为一项铁任务、铁目标，作为检验我们安全生产各项工作是否有成效的铁标准，以改革创新的思路和办法做好安全生产工作。牢固树立企业主体责任是法定、部门监管责任是指令、党政领导责任是天职的理念，严格落实安全生产责任，坚持“党政同责、一岗双责、企业（单位）主责、齐抓共管、失职追责”要求，把安全生产责任逐项分解，逐一落实到部门、企业和单位，落实到岗位、人头和环节，形成完善的责任网络体系。构建风险和隐患双重预防机制，认真落实企业风险防控各项工作，强化政府排查和管控风险责任措施，深入开展隐患排查治理，确保监管到位。深化重点行业领域专项整治，坚持标本兼治、综合治理、系统建设，深入研究各个行业、领域和企业影响安全生产的重大问题，逐项制定具体措施，从根本上解决一批突出问题。推进依法治安，坚持规范化管理，严把安全行业准入关，完善监管执法体制，强化执法检查。加强安全信息化建设，实施一批尾矿库在线视频监控、矿山采空区治理、公路安全生命防护工程等综合治理工程。完善安全工作机制，坚持政府常务会、安全例会逐月研究分析安全生产具体问题，常态化开展宣传培训和警示教育，强化责任追究，促进全市安全生产形势持续稳定好转。

进一步加强和创新社会治理。健全完善“网格化”管理体系，构建城乡社区、社会组织、社会工作联动机制。创新网上信访机制，高度重视征地拆迁、村矿矛盾、医患纠纷等领域的矛盾排查化解，及时妥善处置各类群体性事件。健全应急管理和防灾减灾体系，提高突发事件应急处置能力。深化“平安晋中”建设，完善立体化社会治安防控体系，依法打击违法犯罪活动，有效化解各类社会矛盾，全力维护公共安全和社会稳定。

与此同时，发展妇女儿童、老龄、慈善和红十字会等事业。加强气象、地震、人防、科普、档案等工作。做好民族宗教、外事、侨务、对台等工作。支持国防建设，加强双拥工作。继续做好对口援疆工作。

各位代表，人民群众对美好生活的向往就是我们的奋斗目标。我们要在继续改善人民群众生产生活条件的基础上，更加注重提高人的素质，促进人的全面发展。今年，市政府将再办好十件民生实事：一是实施2900名残疾预防重点干预和残疾儿童抢救性康复项目。二是对4个贫困县建档立卡的农村妇女免费进行“两癌”检查。三是为全市城乡怀孕妇女提供免费产前筛查和诊断服务。四是新建70个农村老年人日间照料中心。五是新增城乡全民健身活动场所100个。六是免费送戏下乡5000场。七是免费送电影下乡3万场。八是实施4548名建档立卡农村贫困劳动力免费职业培训。九是对4个贫困县650名特岗教师和农村教学点青年教师进行三年免费专项培训。十是免费培训乡村医生5000人。

(八)持续提升服务水平，努力在建设有为政府上取得突破。全面加强政府建设，加快职能转变，切实改进作风，不断提升治理体系和治理能力现代化水平。

坚持依法行政。强化法治思维和法治意识，大力推行权力清单和责任清单制度，把政府工作全面纳入法治轨道。坚持科学决策、民主决策、依法决策，实行重大行政决策合法性审查机制、终身责任追究制和责任倒查机制。加强政府智库建设，全面推行法律顾问制度。深入推进相对集中行政许可权改革试点，在市场监管、卫生计生、文化旅游、商务流通、城乡建设、城市管理六个领域全面推开综合行政执法体制改革。探索建立行政执法全过程记录、重大行政执法决定法制审查、行政执法公示等制度。加强行政复议、应诉和法律援助工作，扎实开展“七五”普法，全面推开“两随机、一公开”。 自觉接受人大、政协及社会各界的监督，强化审计监督，实现在一个审计周期内对公共资金、国有资产、国有资源和领导干部履行经济责任情况审计全覆盖。

坚持高效施政。在市县乡三级政府全面推开“13710”工作制度，对省市两级重大决策部署、重要改革事项、重点工作任务，全部及时纳入“13710”督办系统，构建横向到边、纵向到底的执行落实体系。深化“放管服效”改革，完善运行“市民之家”政务服务平台，建立互联网+政务服务体系，加快提升政务服务智慧化水平。深入开展优化投资营商环境专项行动，努力打造高效透明的政务环境和公平公正的营商环境。

坚持担当理政。全面落实《晋中市支持干部改革创新担当作为合理容错纠错实施办法（试行）》，注重过程纠错、及时提醒，为敢于担当的干部担当，为敢于负责的干部负责，营造干事创业的良好环境。健全完善工作推进机制和考核奖惩制度，对不作为、慢作为等行为，严肃追责问责，以问责常态化促进履职、推动落实。

坚持廉洁从政。 认真履行全面从严治党主体责任，深入推进政府系统党风廉政建设和反腐败斗争。坚决把纪律和规矩挺在前面，健全权力关进制度笼子的长效机制。严格执行党内政治生活准则，落实党内监督各项规定。认真落实中央八项规定精神，严格遵守国务院“约法三章”，牢固树立过紧日子思想，厉行勤俭节约，严控“三公”经费，持续狠刹“四风”，促进干部清正、政府清廉、政治清明。

各位代表，实现全面挺进全省第一方阵和全面建成小康社会“两个全面”奋斗目标，是时代赋予我们的历史重任，责任重大，使命光荣。让我们更加紧密地团结在以习近平同志为核心的党中央周围，在省委、省政府和市委的坚强领导下，凝心聚力、开拓进取，忠诚担当、攻坚克难，撸起袖子加油干，扑下身子抓落实，全面完成经济社会发展目标任务，以实际行动和出色成绩迎接党的十九大胜利召开!
